# Cancer Incidence Rates in the US in 2016–2020 with Respect to Solar UVB Doses, Diabetes and Obesity Prevalence, Lung Cancer Incidence Rates, and Alcohol Consumption: An Ecological Study

**DOI:** 10.3390/nu16101450

**Published:** 2024-05-11

**Authors:** William B. Grant

**Affiliations:** Sunlight, Nutrition, and Health Research Center, 1745 Pacific Ave., Suite 504, San Francisco, CA 94109, USA; wbgrant@infionline.net

**Keywords:** alcohol consumption, cancer incidence, diabetes mellitus, diet, ecological study, lung cancer, obesity, solar UVB, USA, vitamin D

## Abstract

This article reports the results of an ecological study of cancer incidence rates by state in the US for the period 2016–2020. The goals of this study were to determine the extent to which solar UVB doses reduced cancer risk compared to findings reported in 2006 for cancer mortality rates for the periods 1950–1969 and 1970–1794 as well as cancer incidence rates for the period 1998–2002 and to determine which factors were recently associated with cancer risk. The cancer data for non-Hispanic white (European American) men and women were obtained from the Centers for Disease Control and Prevention. Indices were obtained for solar UVB at the surface for July 1992, and alcohol consumption, diabetes, and obesity prevalence near the 2016–2020 period. Lung cancer incidence rates were also used in the analyses as a surrogate for smoking, diet, and air pollution. The cancers for which solar UVB is significantly associated with reduced incidence are bladder, brain (males), breast, corpus uteri, esophageal, gastric, non-Hodgkin’s lymphoma, pancreatic, and renal cancer. Lung cancer was significantly associated with colorectal, laryngeal, and renal cancer. Diabetes was also significantly associated with breast, liver, and lung cancer. Obesity prevalence was significantly associated with breast, colorectal, and renal cancer. Alcohol consumption was associated with bladder and esophageal cancer. Thus, diet has become a very important driver of cancer incidence rates. The role of solar UVB in reducing the risk of cancer has been reduced due to people spending less time outdoors, wearing sunscreen that blocks UVB but not UVA radiation, and population increases in terms of overweight and obese individuals, which are associated with lower 25-hydroxyvitamin D concentrations and the generation of systemic inflammation, which is a risk factor for cancer. A dietary approach that would reduce the risk of diabetes, obesity, lung cancer, and, therefore, cancer, would be one based mostly on whole plants and restrictions on red and processed meats and ultraprocessed foods. Solar UVB exposure for a few minutes before applying sunscreen and taking vitamin D supplements would also help reduce the risk of cancer.

## 1. Introduction

Cancer is the second-leading cause of death in the US. In 2021, cancer was responsible for 146.6 deaths per 100,000 inhabitants, whereas heart disease caused 173.8 deaths [1]. In 2024, the American Cancer Society projected 2,001,000 new cancer cases and 612,000 cancer deaths [2]. The probability of developing invasive cancer from birth to death in the 2017–2019 period was estimated at 41.6% for males and 39.6% for females [2]. Cancer is therefore a major health issue in the US.

The major cancer risk factors are reasonably well known. A 2021 American Cancer Society review listed smoking, excess body weight, lack of adequate physical activity, poor diet, alcohol consumption, and infections as major risk factors [3]. A 2019 review by the International Agency for Research on Cancer offered a similar list for the Eastern Mediterranean region. However, for diet, reports list salt intake, red and processed meat, and insufficient fruit and vegetable intake for cancer at various body sites; suboptimal breastfeeding for breast cancer; and air pollution as prominent risk factors for lung cancer [4].

Ecological studies have been used to investigate the role of solar ultraviolet-B (UVB) and vitamin D in reducing the risk of cancer in the US [5,6,7,8] and elsewhere [9,10,11], as discussed in a 2022 review [12]. The brothers Cedric and Frank Garland proposed that vitamin D reduced the risk of colon cancer after seeing data for colon cancer mortality rates in the US in 1974 [5]. A 2002 ecological study [6] used data for the dose of solar UVB at the Earth’s surface in July 1992 obtained from a map in [13] in comparison with cancer mortality rates for white Americans for more than 500 state economic areas as reported in the *Atlas of Cancer Mortality in the United States*, *1950–94* [14]. Significant inverse correlations between solar UVB doses and cancer mortality rates were found for 13 anatomical sites. That work was extended in 2006 by adding several cancer-risk-modifying factors averaged at the state level: alcohol consumption, Hispanic heritage, lung cancer (an index for smoking and diet), poverty, and urban/rural residence. The findings regarding solar UVB were essentially unchanged from the previous study. Another ecological study for non-Hispanic white people for 1993–2002 reported strong inverse correlations between solar UVB doses and cancer incidence and mortality rates for 10 cancers, with weaker evidence for 6 cancers, and inverse relationships that varied by sex for 3 cancers [8]. That study made some adjustments for smoking, outdoor occupation, and atmospheric particulate matter. Thus, in the 1950–2002 period, ecological studies in the US showed significant inverse correlations between solar UVB doses and cancers at many anatomical sites. 

The ecological study approach is similar to satellite measurements of atmospheric constituents and meteorological conditions. They provide a broad overview, helping to guide research and putting findings from other approaches into context. When I worked as an atmospheric scientist for NASA, I flew on many airborne field missions over the Pacific Ocean and elsewhere. In a mission over the US on 13 October 1997, we overflew a case of tropical marine boundary layer and lower tropospheric air masses to the northern midlatitude upper troposphere [15]. The UV differential absorption lidar system I accompanied measured the vertical profile of ozone and aerosols, clearly showing the stratospheric intrusion wrapping around the western edge of the tropical air mass. The Total Ozone Mapping Spectrometer satellite data clearly showed the high ozone content of the intrusion and the low ozone content of the tropical air mass. The GOES-8 satellite image showed the distribution of water vapor in the atmosphere over the US. Backward trajectories of the air masses showed where the air came from. Various in situ instruments inside the aircraft measured concentrations of various molecular species in the tropical air mass on 13 October and in other air masses on subsequent flights. Combining the data from all of these and other sources enabled a more complete understanding of the event than possible using only one source. 

Other approaches used to examine the role of vitamin D in the risk of cancer include observational studies such as prospective cohort studies, randomized controlled trials (RCTs), and studies of mechanisms. A 2022 review summarized what had been found from the four types of study by that time [12]. Geographical ecological studies reported findings regarding indices for solar UVB dose and incidence and mortality rates for 32 types of cancer. The advantages of ecological studies include that since they are based on published data sets, little effort is required to perform the analysis. In addition, since they use large data sets, results can be obtained for many types of cancer, which is generally not possible in observational studies or RCTs. In addition, ecological studies can be used to evaluate the role of various risk-modifying factors on the risk of cancer, as carried out in the present study. Limitations of ecological studies include that the data are averaged for population groups and, thus, may not apply to those who develop cancer. Also, some of the important risk-modifying factors may not be included in the analysis. Meta-analyses of observational studies of the incidence risk of individual cancer sites related to serum 25(OH)D concentration have been reported for 11 types of cancer. As discussed in the 2022 review [12], an important limitation of observational studies is that the apparent beneficial effect of higher serum 25-hydroxyvitmin D [25(OH)D] is inversely correlated with follow-up time. A number of RCTs have examined the effect of vitamin D supplementation on cancer incidence and/or mortality rates. However, methodological problems with design, conduct, and analysis have limited the findings from these and other vitamin D RCTs [16,17,18]. The 2022 review included 12 pages regarding the mechanisms whereby vitamin D reduces the risk of cancer. Thus, as discussed for atmospheric measurements, using findings from several types of studies provides a better understanding of the role of solar UVB exposure and vitamin D in risk of cancer than using results from only one type of study such as RCTs.

The standard hierarchy of evidence generally followed in medicine is a pyramid that starts with in vitro studies at the bottom, progressing through animal studies, ecological studies, cross-sectional studies, case–control studies, cohort studies, RCTs, and, at the top, meta-analyses of RCTs [19]. A recent article suggested replacing the pyramid with a wheel [20]. The starting pyramid had background information and expert opinion at the lower rung, then proceed through case–control studies, cohort studies, RCTs, critically appraised topics and articles, and systematic reviews at the top. The rungs in the pyramid were mapped into non-systematic reviews, clinical studies, and structured analyses. Mechanisms of action was a fourth element of the wheel. The wheel approach seems to be a much better approach for evidence for sun exposure and vitamin D and health outcomes since it encompasses much more information than, for example, RCTs. 

A chance perusal of cancer incidence data for 2016–2020 in the US posted by the Centers for Disease Control and Prevention (CDC) [21] showed that the distribution of cancer incidence had changed in several important ways since 2002. The main difference was that cancer incidence rates in the southeastern states were much higher than before. In addition, the strong inverse correlations between solar UVB doses and cancer rates were either not as strong or absent for several cancer sites. Thus, this new ecological study was initiated. The goals were to determine the extent to which solar UVB exposure reduced cancer risk in the recent past and which factors seem to be important cancer risk factors at present.

## 2. Materials and Methods

Cancer incidence data (cases/100,000/year) were obtained from the CDC’s Cancer Statistics At a Glance website [21]. The data are age-adjusted to the 2000 U.S. standard population [22]. Those statistics include cancer registry data from the CDC’s National Program of Cancer Registries [23] and the National Cancer Institute’s Surveillance, Epidemiology, and End Results (SEER) Program [24]. The SEER Program is an authoritative information source on cancer incidence and survival in the United States. SEER collects and publishes cancer incidence and survival data from population-based cancer registries covering about 48.0% of the US population. SEER coverage includes 42.0% of white people, 44.7% of African Americans, 66.3% of Hispanic people, 59.9% of American Indians and Alaska Natives, 70.7% of Asian people, and 70.3% of Hawaiian/Pacific Islanders. Data were available for 2016–2020 with data available by race/ethnicity, sex, and anatomical site. No data were available for Indiana, Nevada, and North Dakota. The data values appear to have low 95% confidence intervals (95% CI). For example, for pancreatic cancer for males, in Nebraska, the rate is 15.3 (95% CI, 14.2–16.4) cases/100,000/year. For CRC for males, in Nebraska, the rate is 44.7 (95% CI, 42.9–46.6) cases/100,000/year. Only data for non-Hispanic white people (NHW) were used in this study. While data are available for other races/ethnicities, solar UVB has less impact on people with darker skin, making it more difficult to perform an ecological study, as shown in a 2006 ecological study of cancer mortality rates for African Americans with respect to solar UVB doses [25]. In addition, race/ethnicity-specific data for other cancer risk-modifying factors would not be easy to obtain. Table 1 lists the cancers considered in this study and the mean rates for the period 2016–2020.

The risk-modifying factors included in this ecological study were based on what was used in a previous ecological study of cancer mortality rates in the US [7], indicated as being correlated with cancer incidence rates after looking at a number of recent maps on the CDC’s website. These factors include data averaged at the state level for solar UVB dose, lung cancer incidence rates, diabetes mellitus (DM) rates, obesity rates, and ethanol consumption amounts. Several factors included in the 2006 study are not included in the present study since they no longer seem to be relevant: socioeconomic status, urban/rural residence, and Hispanic prevalence.

Solar UVB dose is used as the index of serum 25(OH)D concentration. Solar UVB dose data were obtained from a map [13] based on data obtained with NASA’s Total Ozone Mapping Spectrometer satellite instrument [26]. Data in this map were carefully digitized for about 500 state economic areas corresponding to maps in the Atlas of Cancer Mortality Rate [14], then averaged by state, and weighted by population densities. 

Table 2 shows digital values determined from a map [13]. Data for Alaska and Hawaii were omitted because those two states are at the extreme latitudes of the US and, as a result, are not representative of solar UVB’s effect on cancer incidence due to either vitamin D supplementation or very high UVB doses. Wintertime serum 25(OH)D concentrations are about 60–70% of summertime values [27,28]. An important reason is that 25(OH)D stored in muscles is released into the blood in a manner that keeps serum 25(OH)D concentrations reasonably high in the absence of vitamin D production or oral intake [29,30]. 

Lung cancer incidence rates were included as one determinant of cancer incidence. Though lung cancer is normally thought of as being from smoking, air pollution also plays a role [31,32], as does diet [33]. Thus, this study used lung cancer incidence in 2016–2020 for males and females as an index of air pollution, diet, and smoking. Although indices for the three factors might be available, using lung cancer incidence is simpler.

Because rates of DM and obesity have increased considerably in the US and are highest in the southeast, this study includes data for the prevalence of both conditions by state. Data for DM came from the CDC. The data were for the prevalence of DM for non-Hispanic white people (NHWs) older than 18 years averaged over 2016–2020 [34]. The data were obtained by the Behavioral Risk Factor Surveillance System (BRFSS) [35], the nation’s premier system of health-related telephone surveys that collect state data about US residents regarding their health-related risk behaviors, chronic health conditions, and use of preventive services. The BRFSS completes more than 400,000 adult interviews each year. An estimate of the uncertainty of the data can be made using data from Nebraska. For 2019, the prevalence was 8.4 (95% CI, 7.8–9.0)%. Data for five years was used in the analysis, thereby reducing the 95% CI to about 0.6 × 0.6 = 0.4%.

Data for obesity came from the CDC [36], obtained by the BRFSS [35]. Data for NHWs for males and females combined were averaged for 2017–2019. Data were available in three-year averages. It was decided to use a value from the middle of the range of cancer data rather than combine data from two three-year periods. Obesity prevalence for 2015–2017 was compared to that for 2017–2019. The equation for the regression fit to the data was obesity (2017–2019) = 1.07 + 1.01 × obesity (2015–2017), *r* = 0.98. Thus, it appears that little is lost by using the single three-year averaged data. 

Data for the percentage of the population in urban and rural regions by state were obtained from the US Census Bureau [37]. Because no cancer rates were significantly associated with urban/rural residence, those results are not presented.

Data for alcohol consumption is per capita gallons of ethanol consumed by state for 2016 [38] and 2022 [38]. The data from the Vinepair website were based on data from the National Institute on Alcohol Abuse and Alcoholism. The data were more readily accessible from Vinepair than from the US government site. A comparison of the data for the two years found that consumption rates in 2016 were much higher than those for 2022 for three states: Idaho, South Dakota, and Washington. When the 2016 consumption rates for those three states were omitted, the equation for the regression fit to the data was consumption (2022) = −0.032 + 1.05 × consumption (2016), *r* = 0.98. Thus, the consumption values for 2022 were used for those three states. Since there was very little change between 2016 and 2022, it seems that the 2016 consumption rates are appropriate for use, in part since alcohol consumption prior to cancer incidence is likely more important than near the time of cancer diagnosis.

Data were analyzed using SigmaStat 4.0 (Grafiti, Palo Alto, CA, USA). Data plots were made using KaleidaGraph Version 4.5.4 (Synergy Software, Reading, PA, USA).

## 3. Results

**Figure 1:**
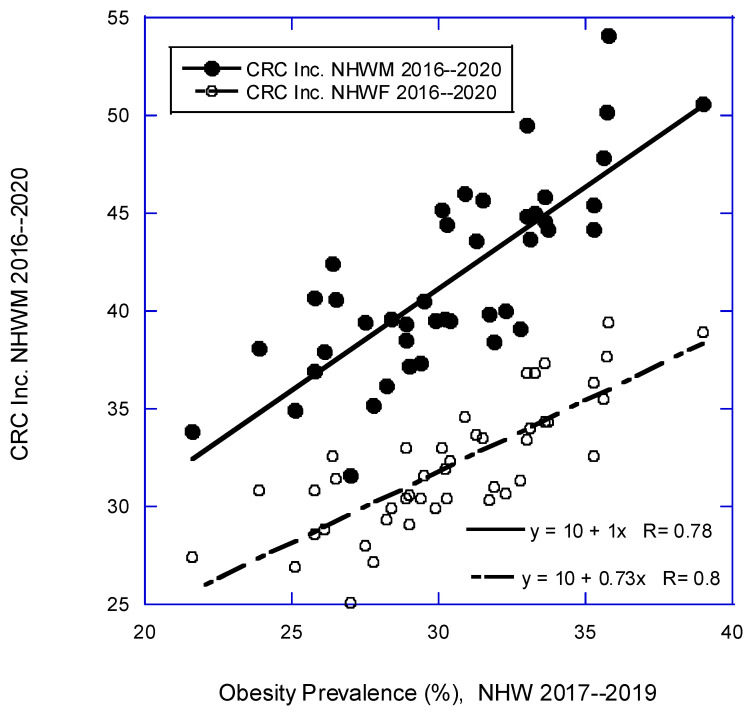
Scatter plot of CRC incidence for NHW males and females [21] vs. obesity prevalence (%) for NHW men and women in the period 2017–2019 [36].

**Figure 2:**
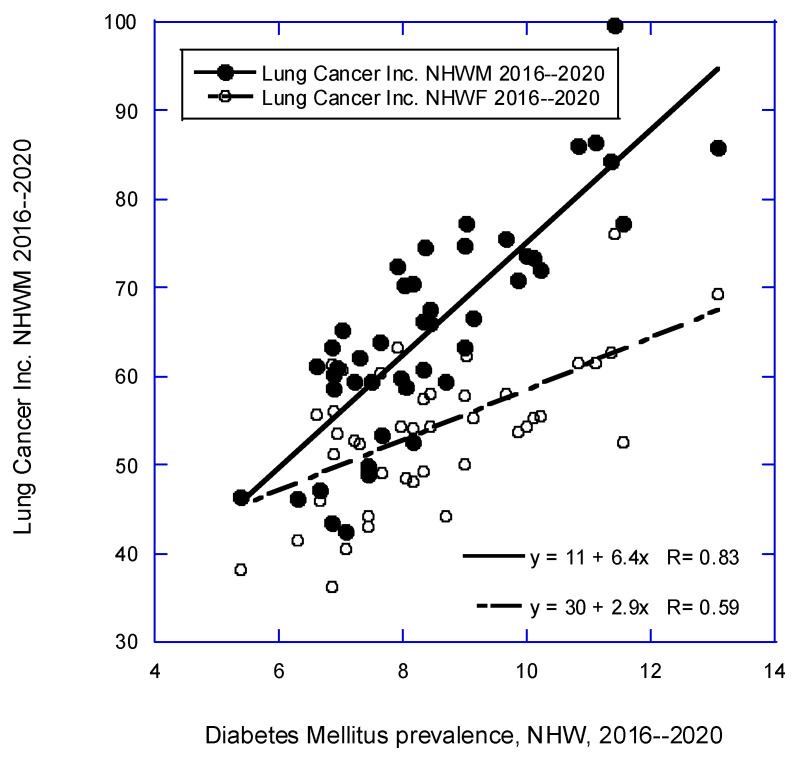
Scatter plot for lung cancer incidence rates by state for NHW males and females [21] vs. diabetes mellitus prevalence (%) for 2016–2020 [34].

**Figure 3:**
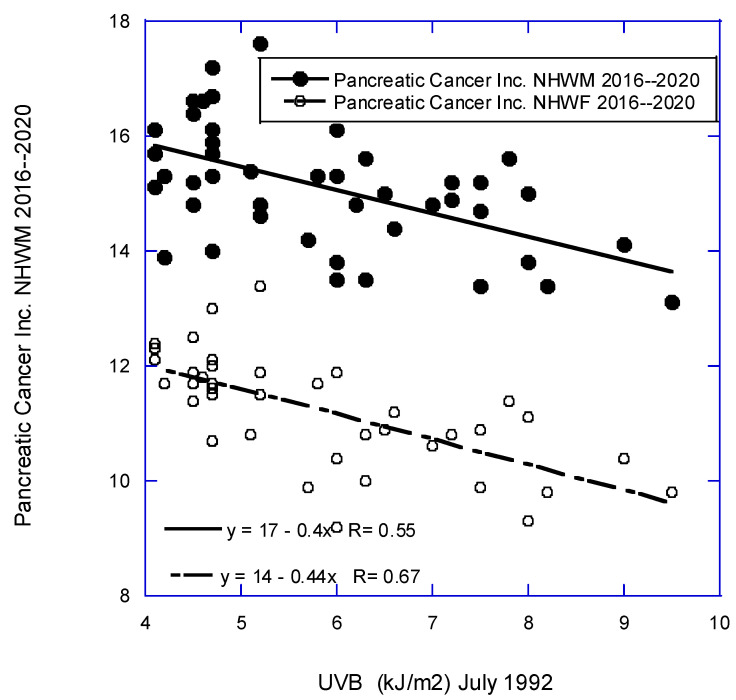
Scatter plot of pancreatic cancer incidence rates by state for the period 2016–2020 [21] vs. solar UVB doses for July 1992 [26].

Figure 1, Figure 2 and Figure 3 are scatter plots of cancer incidence rates with respect to three of the factors used in this study. Figure 1 shows the correlation for CRC for females and males with respect to obesity rates for NHW people in 2017–2019 [36]. Figure 2 shows lung cancer incidence rates for females and males with respect to DM rates from 2016 to 2020 [34]. Figure 3 shows pancreatic cancer incidence rates for females and males with respect to solar UVB doses for 1992 [26]. These plots indicate that the various factors used in this study have high correlations with various types of cancer.

Table 3 shows the cross-correlation coefficients for the factors used in this ecological study. Factors that are significantly correlated should not be used in the same analysis. Instead, such factors can be used sequentially to see which results in a higher correlation with cancer incidence.

Table 4 and Table 5 show the important statistical analyses from this ecological study. Solar UVB is significantly associated with reduced incidence of bladder, brain (males), breast, corpus uteri, esophageal, gastric, non-Hodgkin’s lymphoma, pancreatic, and renal cancers. Lung cancer was the only risk factor found for laryngeal cancer. However, lung cancer also was significantly associated with colorectal and renal cancers. Diabetes also was significantly associated with breast, liver, and lung cancers. Obesity prevalence was significantly associated with breast, colorectal, and renal cancers. Alcohol (ethanol) consumption was associated with bladder and esophageal cancers. The associations of diabetes and obesity prevalence with incidence rates for various cancers can be due both to the direct effects of diabetes and obesity as well as the effects of underlying causes such as lifestyle including diet. See the discussion section for more details.

Table 6 compares the results of this ecological study with the cancer incidence rate ecological study based on data from 1998 to 2002 by Boscoe and Schymura [8] and the ecological study based on cancer mortality rate data for 1950–1969 and 1970–1994 by Grant and Garland [7]. All three studies reported inverse correlations between solar UVB and cancer incidence and mortality rates for bladder, corpus uteri, esophageal, gastric, pancreatic cancer, and non-Hodgkin’s lymphoma (NHL). Brain cancer also was inversely correlated with solar UVB doses in the Boscoe and Schymura study [8]. Cancer sites inversely correlated with solar UVB in one or both of the earlier studies but no longer so associated are colorectal, laryngeal, ovarian, renal cancer, Hodgkin’s lymphoma, and myeloma. The discussion section describes the implications for understanding vitamin D’s role in reducing the risk of cancer incidence and mortality rates.

## 4. Discussion

An analysis of the state of US health from 1990 to 2016 showed that the major risk factors for disability-adjusted life-years (DALYs) by state included, in descending order, tobacco use, high body mass index (BMI), dietary risks, alcohol and drug use, high fasting plasma glucose, high systolic blood pressure, high total cholesterol, impaired kidney function, occupational risks, air pollution, and low physical activity [39]. The findings in this ecological study are generally consistent with the order of those factors, especially when considering that several are related to diet.

### 4.1. Diet

A large body of peer-reviewed journal literature reports that diet is a major risk-modifying factor for lung cancer. A case–control study in Texas involving 2139 non-small-cell lung cancer (NSCLC) cases who completed food frequency questionnaires for the year before cancer diagnosis were compared with 2163 matched controls [40]. Participants were from many races/ethnicities, which the analysis did not consider. Three dietary patterns were evaluated: fruits and vegetables, American/Western, and Tex-Mex. The multivariable-adjusted odds ratio (aOR) for NSCLC for quantile 5 versus quantile 1 of fruits and vegetables was 0.68 (95% CI, 055–0.85); for American/Western, 1.45 (95% CI, 1.18–1.78); and Tex-Mex, 0.45 (95% CI, 0.37–0.56). For never-smokers, the aOR for fruits and vegetables was 0.99 (95% CI, 062–1.58); for American/Western, 2.01 (95% CI, 1.25–3.24); and Tex-Mex, 0.50 (95% CI, 0.32–0.78). The aORs for former smokers and current smokers were similar to the results for all participants.

In this ecological study, the association with lung cancer for diabetes was stronger than for obesity. Obesity is not considered as strong a risk factor for lung cancer as is waist circumference [41]. The same holds true for diabetes [42,43].

Obesity has been identified as a risk factor for several cancers. A 2013 review listed six cancers caused by obesity: breast, colorectal, endometrial, pancreatic, prostate, and renal cell carcinoma [44]. The mechanisms for the three cancers that this study supports are, for breast cancer, a decrease in sex-hormone-binding globulin and hormonal factors; for colorectal cancer, steroid hormones and chronic inflammation; and for renal cell carcinoma, an increased level of estrogen. A 2016 review also listed high BMI as a modifiable risk factor for breast cancer among white women in the US [45]. A 2019 review listed obesity, insulin resistance, and adipokine aberrations as being jointly linked to cancer risk [46]. Adipose tissue increases in obese individuals and results in the production of adipokines, which trigger low-grade inflammation and insulin resistance [47]. Also, the altered gut microbiome contributes to inflammation and carcinogenic products [46].

Obesity rates have risen in the US recently. Obesity rates for NHW adult men aged 20 years or older rose from a mean of 26.6% in 1999–2000 to 38.0% in 2015–2016 according to National Health and Nutrition Examination Survey (NHANES) data from 1999–2016 [48]. For NHW adult women, the corresponding values were 33.5% and 41.5%.

A recent article [49] suggested following the Mediterranean diet [50] to manage obesity. The main guidelines are low intake of red and processed meat and refined sugar; moderate intake of low-fat dairy products, poultry, fish, and red wine; and high intake of virgin olive oil, nuts, fruit and vegetables, legumes, and unrefined whole grains. Those recommendations are in general agreement with the findings in a 2023 Harvard cohort study [51].

Good evidence exists suggesting that diet affects the risk of colorectal cancer (CRC). A 2015 article from the Adventist Health Study 2 reported that in a prospective observational study of vegetarians and nonvegetarians, the adjusted hazard ratio for CRC was 0.78 (95% CI, 0.64–0.95) [52]. In an analysis of food intake based on data from NHANES, 2007–2010, and the USDA Food Patterns Equivalents Database, 2007–2010, vegetarians consumed 1862 kcal, whereas nonvegetarians consumed 2058 kcal [53]. A 2019 review listed the driving forces behind the increase in CRC as obesity, a sedentary lifestyle, and red meat, alcohol, and tobacco consumption.

Studies of changes in cancer rates in countries that experienced the nutrition transition to the Western dietary pattern in the past half-century offer more support for diet’s role in cancer risk. For example, an analysis of data from China, Hong Kong, Japan, Korea, and Singapore showed remarkable increases in mortality rates of breast, colon, and prostate cancers and precipitous decreases in mortality of esophageal and gastric cancers [54]. Those results are consistent with findings in the present ecological study for breast and colorectal cancer (with obesity as a risk factor). They also are probably consistent with the findings for esophageal and gastric cancers in that neither diabetes nor obesity was found to be a risk factor. In an ecological study involving eight countries—Brazil, China, Cuba, Egypt, India, Nigeria, the Republic of Korea, and Sri Lanka—20-year increases in dietary supply of energy and animal fat were significantly associated with increases in Alzheimer’s disease and dementia rates [55].

Diet is an important risk factor for type 2 diabetes mellitus (T2DM). A 2023 article reported findings from a cohort study involving 205,852 health professionals monitored for up to 32 years [51]. The participants completed food frequency questionnaires every 4 years and described changes in health status. The study included 37 food groups. The data were then correlated with various dietary patterns such as DASH and an American version of the Mediterranean diet. In addition, two empirical dietary patterns were developed: the reversed empirical dietary index for hyperinsulinemia (rEDIH) and the reversed empirical dietary inflammatory pattern (rEDIP). Both insulin resistance and systemic inflammation, often associated with obesity, are significant risk factors for many diseases, including T2DM [56,57] and cancer [58]. The rEDIH and rEDIP dietary patterns had the strongest inverse correlations with T2DM. For the highest decile compared with the lowest decile, the multivariate-adjusted risk for T2DM was 0.36 (95% CI, 0.35–0.37) for rEDIH and 0.38 (95% CI, 0.37–0.40) for rEDIP. When BMI was added, the values changed to 0.57 (95% CI, 0.54–0.59) and 0.57 (95% CI, 0.55–0.59), respectively. The food groups most strongly associated with high risk of disease were red meats, processed meats, energy drinks, french fries, and refined grains, whereas the food groups most strongly associated with reduced risk included coffee, leafy green vegetables, whole grains, fruit, dark-yellow vegetables, and salad dressing.

Further evidence shows that red meat and processed meat are important risk factors for cancer. A case–control study in Uruguay reported that both types of meat significantly correlated with the incidence of NHL [59]. A 2015 review showed that 9 of 10 meta-analyses reported red and/or processed meat to be significantly correlated with the risk of CRC [60]. A 2021 meta-analysis of prospective studies showed red and/or processed meat to be significantly directly correlated with the incidence of breast, colon, colorectal, lung, rectal, and renal cancers [61]. It has been proposed that intestinal microbiota helps mediate the link between red/processed meat consumption and the risk of colon cancer [62].

A study conducted from 2003 to 2007 reported that participants consuming the highest quartile of the Southern dietary pattern (characterized by added fats, fried food, eggs, organic and processed meats, and sugar-sweetened beverages) experienced an adjusted 37 (95% CI, 1–85)% higher risk of coronary heart disease than those in the lowest quartile [63].

T2DM was treated with a high-fiber, low-fat, plant-predominant diet in Virginia, USA [64], consisting of 40% vegetables, 20% beans, 15% whole grains, 10% fruits, 10% seeds/nuts, and 5% egg whites and nonfat milk. The mean BMI immediately before the lifestyle change was 33 (SD = 6), dropping to 30 (SD = 6) after 6 months. Fasting glucose decreased from 140 mg/dL (SD = 40 mg/dL) to 110 mg/dL (SD = 20 mg/dL). Twenty-two of fifty-nine patients achieved T2DM remission.

An important but relatively little-known fact about the US food supply is that concentrations of essential minerals have been decreasing. A 2002 review outlined the evidence that mineral deficiencies are a major cancer risk [65]. A 2007 article reported the weighted average depletion of essential minerals in the US food supply [66]. It was based on data for cheeses, dairy, and meat from 1940 to 2002 and on fruits and vegetables from 1940 to 1991. Depletions were 29% for calcium, 62% for copper, 37% for iron, 19% for magnesium, 15% for potassium, and 34% for sodium. The reasons for the decreases include acid deposition [67], extraction by harvested agriculture products, and widespread use of glyphosate fertilizer. Glyphosate fertilizer reduces seed and leaf concentrations of important minerals [68]. It decreases mycorrhizal colonization and adversely affects plant–soil feedback [69]. The fertilizer adversely affected soil bacteria, soil chemistry, and mycorrhizal fungi during the restoration of a Colorado grassland [70].

A quick search of publications regarding mineral intake and risk of cancer found that higher iron and zinc intake was associated with a reduced risk of lung cancer in a 22-year study [71]. Higher combined mineral intakes of 11 minerals were inversely correlated with the risk of CRC in postmenopausal women in a prospective study conducted in Iowa [72]. A 2022 review provides a recent overview of the importance of minerals in cancer risk [73].

Minerals are also important for reducing the risk of T2DM. A 2020 review outlines the role of minerals and trace elements in reducing the risk of insulin resistance and T2DM [74]. Studies in China found that copper and zinc concentrations were inversely correlated with T2DM [75], and that while iron was directly correlated with T2DM this association was reduced to a non-significant correlation with higher concentrations of antioxidant minerals including chromium, copper, magnesium, selenium, and zinc [76].

A 2022 review of spatial-temporal patterns of incidence, mortality, and attributable risk factors for T2DM from 1990 to 2019 among 21 world regions showed high BMI (52%), ambient particulate matter (14%), smoking (10%), and secondhand smoke (9%) to be the major contributing factors to T2DM disability-adjusted life-years [77].

### 4.2. Cigarette Smoking

Cigarette smoking is, of course, an important risk factor for lung cancer as well as several others. A 2002 review listed cancers for which tobacco smoking was considered a risk factor for mortality: cervical, esophageal, laryngeal, lung trachea and bronchus, oral cavity; pancreatic, renal, and urinary bladder [78]. A 2001 review of observational studies of cigarette smoking and the risk of colorectal adenoma and CRC showed strong support for causality [79]. Smoking can take 3–4 years to result in CRC. That study suggested that smoking could account for 20% of CRC deaths in the US. The present study shows that only four of those cancers were related to lung cancer: CRC, esophageal, laryngeal, and renal. However, lung cancer was significantly correlated with all cancers less lung cancers for both males and females.

A 2014 article presented maps of cigarette smoking for 1996 and 2012 for males and females in US counties [80]. Smoking rates decreased considerably between the two periods. Rates were higher for males than for females. States in the continental US with the highest smoking rates were in the southeast, from Mississippi to West Virginia.

### 4.3. Particulate Air Pollution

Particulate air pollution (PM_2.5_) concentrations in the US are mostly higher in the eastern US but also in California and in and near Idaho [81,82]. A 2009 study based on MODIS satellite data of aerosol optical depth in 2003 and 2004 reported a high correlation of the aerosol optical depth with age- and race-standardized mortality rates of chronic coronary heart disease (β_PM2.5_ = 0.80; posterior 95% Bayesian credible interval, 0.39–1.23) [81]. For a cohort of 44,610 individuals in the southeast, a 2021 article based on correlations between satellite data and incident cardiovascular disease reported a 13.4% increase in risk with exposure to unhealthy levels of PM_2.5_ at the time of enrollment [83].

### 4.4. Solar UVB and Vitamin D

The role of solar UVB and vitamin D in reducing the risk of cancer incidence and mortality rates was reviewed in 2022 [12]. Supporting evidence comes from various studies stretching back to 1936 when researchers recognized that sun exposure can cause skin cancer but reduce the risk of internal cancers [84]. As discussed, ecological studies in the US have yielded good evidence that solar UVB reduces the risk of incidence and mortality rates for many cancers [7,8]. Similar results have been reported from China [9], Russia [10], and Nordic countries [11]. No factor other than vitamin D production has been proposed to explain the inverse correlation of solar UVB doses with cancer risk.

Solar UVB doses might have had lower correlations with cancer incidence rates in the 2016–2020 period than in earlier periods in the US for several reasons:

*Reduced time spent in the sun when vitamin D can be produced.* Because solar UVB reaching Earth’s surface increases as the solar elevation angle increases [85], it is generally recognized that vitamin D can be made effectively when the angle is greater than about 45°.

*Wearing sunscreen or sunblock.* Many cosmetics now contain sunscreen [86].

*Increased prevalence of overweight and obesity.* An inverse correlation generally exists between serum 25(OH)D concentration and weight or BMI. A meta-analysis reported that“The prevalence of vitamin D deficiency was 35% higher in obese subjects and 24% higher than in the overweight group” [87]. Also, obesity is associated with increased systemic inflammation, thereby increasing the risk of cancer [58]. This mechanism could possibly explain why solar UVB and vitamin D are less effective in reducing the risk of cancer as found in the VITAL trial [88]. In that trial, participants in three BMI categories, <25 kg/m^2^, 25 to <30 kg/m^2^, and >30 kg/m^2^ had the same increase in 25(OH)D concentration, ~12 ng/mL, but only those with BMI <25 kg/m^2^ had a significantly reduced risk of cancer incidence.

Prospective cohort studies of cancer incidence with respect to serum 25(OH)D at the time of enrollment have shown inverse correlations for bladder, breast, colorectal, liver, lung, and renal cancers (Table 5 in [12]). An important problem in conducting meta-analyses of such studies is to properly account for changes in serum 25(OH)D since enrollment [89]. As shown in Figure 1 in [12], a nearly linear change occurs in the odds ratio with follow-up time for CRC. When properly accounted for, the relative risk (RR) drops to 0.74 for men and 0.77 for women. That finding differs from what was reported in the 2019 article by McCullough and colleagues in which it was reported that men had a considerably lower reduction of CRC than women [90].

Randomized controlled trials (RCTs) offer less support for vitamin D’s role in reducing the risk of cancer incidence and death. The main reason is that most RCTs are based on guidelines for pharmaceutical drugs, not for nutrients. In drug trials, the only source of the drug is the trial itself, participants in the control arm are given a placebo, and results are analyzed on an intention-to-treat basis. That approach is not appropriate for vitamin D because vitamin D is available from other sources besides the trial, and cancer outcomes are related to serum 25(OH)D concentrations, not vitamin D doses. Heaney outlined guidelines for nutrients in 2014 [91]. The important guidelines include that serum 25(OH)D concentrations should be measured before enrollment and that people with low values should be included in the trial; that the vitamin D dose should be large enough to raise serum 25(OH)D concentrations enough to significantly reduce the risk of the health outcome of interest; and that achieved serum 25(OH)D concentration should be measured and used in analyzing the results. A 2022 review further discusses the topic [18].

An example of the importance of following such guidelines is found in the prediabetes-to-diabetes trial conducted by Tufts University gave people in the treatment group 4000 IU/d of vitamin D_3_ [92]. When results were analyzed by intention to treat, no significant difference in progression to diabetes was apparent between the treatment and placebo arms. However, when results were analyzed by achieving 25(OH)D concentration in the treatment group, researchers found that participants in the vitamin D treatment arm who had 25(OH)D concentrations above 50 ng/mL during the trial had a hazard ratio for progression to diabetes of 0.29 [95% CI, 0.17–0.50] compared with those who maintained a level of 20–30 ng/mL [93].

The largest vitamin D–cancer RCT conducted was Harvard Medical School’s **VITamin D** and **OmegA-3 TriaL** (**VITAL**) [88]. More than 25,000 participants were enrolled, including more than 5000 African Americans. Participants in the treatment arm were given 2000 IU/d of vitamin D_3_, but participants in both the treatment and placebo arm were permitted to take up to 600 or, if older than 70 years, 800 IU/d of vitamin D_3_. Nearly 17,000 participants submitted serum 25(OH)D concentrations near the time of enrollment. The mean 25(OH)D concentration of those in the treatment arm was near 31 ng/mL. The median follow-up time was 5.3 years. The abstract reported that vitamin D did not significantly reduce the risk of cancer incidence but seemed to modestly reduce the risk of cancer mortality rates. However, the article reported that the HR for cancer incidence for those with BMI < 25 kg/m^2^ was 0.76 (95% CI, 0.63–0.90). In addition, the HR for African Americans was 0.77 (95% CI, 0.59–101), which barely failed the *p* = 0.05 test of significance. Those results were not discussed in press conferences regarding the findings, and so busy physicians who read only the abstract were unaware of those results.

The mechanisms whereby vitamin D reduces the risk of cancer incidence and mortality rates are well known [12]. Vitamin D reduces cancer risk by surveilling cells and regulating apoptosis, differentiation, and progression. Vitamin D reduces progression by reducing angiogenesis around tumors and reduces metastasis by regulating concentrations of MMP-9. Matrix metalloproteinases (MMPs) are zinc-dependent proteolytic metalloenzymes, of which MMP-9 is one of the most complex. MMP-9 can degrade the components of the extracellular matrix [94]. Many more mechanisms also exist. For example, a recent article reported that vitamin D reduces the risk of cancer by regulating gut-microbiome-dependent cancer immunity [95].

Researchers recently determined that patients with digestive tract cancers who are p53-immunoreactive have a much better survival rate with vitamin D supplementation [96]. Holick wrote the accompanying editorial, pointing out its importance in treating cancer [97]. That finding seems likely to apply to all types of cancer.

Several reviews make recommendations regarding vitamin D supplementation. A 2024 review outlined the rationale for supplementing with 2000 IU/d (50 μg/d) of vitamin D_3_ for most adults [98].

### 4.5. Changes in Cancer Incidence Rates

At this point, it is interesting to examine what changes have been found regarding cancer incidence rates in the US. The source for such data is Cancer Statistics, 2024 [2]. The calculations were made using the midpoints for the 1970–1994 and 2016–2020 incidence rate figures. The results are shown in Table 7. Interestingly, rates tended to fall for men but to increase for women except for CRC. Lung and bronchus cancer incidence rates for men peaked in ca. 1985 for men and 2005 for women [2]. A 2014 article reported that total cigarette smoking among men fell from 1996 to 2012 by 0.9% per year for men and 0.6% for women [80]. A meta-analysis of 77 articles found that active smoking increased the risk of breast cancer (OR -= 1.15 [95% CI, 1.11–1.20]) [99].

The fact that melanoma incidence rates have risen is very interesting. The risk of melanoma is related more to solar UVA (315–400 nm) than solar UVB (290–315 nm). This was demonstrated in an ecological study of melanoma mortality rates for men in 45 countries [100]. It was proposed in 1993 that the increased use of sunscreens explained the increasing trends of melanoma incidence from 1935 to 1985 [101]. It was noted that a common ingredient of sunscreens, para-aminobenzoic acid, had peak blockage of UVB at 290 nm, falling to zero blockage at 330 nm, thereby blocking very little solar UVA radiation. A meta-analysis of sunscreen use found a significant increase in melanoma for sunscreen use in countries north of 40° N latitude (OR = 1.6 [95% CI, 1.3–−1.9]) but not south of 40° N latitude (OR = 0.7 [95% CI, 0.4–−1.0]) [102]. The evidence that vitamin D reduces the risk of melanoma is very strong [103]. Thus, it is recommended that people using sunscreen spend a few minutes in the sun before applying sunscreen [104]. This analysis provides strong support for the hypothesis that an important reason why the effect of solar UVB dose was diminished in the 2016–2020 period compared to the 1950–1969 and 1970–1994 periods.

### 4.6. Public Health Implications

The findings in this ecological study could play an important role in public health policy and cancer prevention. The results indicate that dietary risk factors for lung cancer, obesity, T2DM, and solar UVB exposure/vitamin D are important risk-modifying factors for cancer incidence. Government agencies and disease organizations are important determinants of public health policies for diet and vitamin D. This section will compare public health policies from those sources with current scientific evidence.

Solar UV and vitamin D policies are considered first. The National Institutes of Health, Office of Dietary Supplements, states the following in their report on vitamin D for health professionals: “Although 25(OH)D functions as a biomarker of exposure, the extent to which 25(OH)D levels also serve as a biomarker of effect on the body (i.e., relating to health status or outcomes) is not clear. Researchers have not definitively identified serum concentrations of 25(OH)D associated with deficiency (e.g., rickets), adequacy for bone health, and overall health”. That statement is based on a report from 2011 [105], prepared before 14 years of additional research. It is in need of an update. While the American Cancer Society has good information regarding several ways to reduce the risk of cancer, it recommends staying safe in the sun through the use of sunscreen, etc., without mentioning that UVB, through the production of vitamin D, can reduce the risk of cancer so that regular sunscreen use should be accompanied by regular vitamin D supplementation [106]. The US Preventative Services Task Force recommended in 2021 that adults should not be screened for vitamin D deficiency [107]. It concluded “Among asymptomatic, community-dwelling populations with low vitamin D levels, the evidence suggests that treatment with vitamin D has no effect on mortality or the incidence of fractures, falls, depression, diabetes, cardiovascular disease, cancer, or adverse events”—a statement that clearly needs to be updated.

Red meat and processed meat are among the most important risk factors for T2DM [51,108,109]. There is also good evidence that T2DM can be reversed through a plant-based diet [110]. A 2020 consensus report by the American Diabetes Association and several other organizations recommended that patients work with dieticians but did not make any dietary recommendations [111]. Disease organizations, such as the American Diabetes Association, could do more to inform people how to prevent and reverse DM.

Meat and ultraprocessed food (UPF) are important risk factors for obesity. A 2009 study found that people in the upper quintile of meat consumption consumed 700 more kCal/day than those in the lowest quintile [112]. A 2022 paper opined that the major cause of the obesity epidemic in the US is UPF [113]. The author noted that healthy foods cost more than unhealthy foods, due perhaps to increased subsidies to farmers beginning in the 1970s, leading to more production of foods that could be made into UPF and sold cheaply. A 2023 meta-analysis found that a 10% increase in UPF consumption was associated with a 6% increased risk of obesity [114]. UPF consumption is also an important risk factor for T2DM [115].

The Dietary Guidelines for Americans, 2020–2025 recommended keeping added sugars to less than 10% of total calories/day, saturated fat to less than 10% of calories/day, and sodium to less than 2300 mg/day [116]. There was a recommendation to minimize foods with added sugars, UPF, processed meats, foods high in salt, alcoholic beverages, and toxic oils. However, there was no recommendation to limit red meat consumption. A 2022 article noted that the vast majority of dietary guideline committee members had at least several conflicts of interest directly relevant to their work on the scientific report such as working for, or having research funded by, food and/or pharmaceutical companies [117].

Thus, at present, the effort to inform the general public on how to improve their health through dietary choices, vitamin D supplements, and sensible sun exposure seems to fall mainly on non-profit organizations that recommend supporting such measures.

### 4.7. Strengths and Limitations

The strengths of this study include that it provides information regarding risk-modifying factors for cancer in the US in the period 2016–2020. It includes data for three factors related to diet, DM and obesity prevalence, and lung cancer incidence rates in addition to solar UVB doses and alcohol consumption rates. It shows that dietary factors have become comparable if not stronger risk-modifying factors than solar UVB exposure. The ecological study approach is similar to satellite measurement of air quality, which has provided much useful information for health studies [118]. Among other things, it shows the regions of greatest and least risk and provides data that would be very time-consuming to obtain from observational studies.

The limitations include that further risk-modifying factors were not included such as food group consumption patterns, cigarette smoking rates, particulate matter pollution concentrations, and serum 25(OH)D concentrations. In addition, the data sets are approximations based on data for only a fraction of the population for cancer incidence rates and the other factors used. Also, the data for the various factors are for the population as a whole, not for those who developed cancer. Also, the UVB data are from one month in July 1992 and do not adjust for aerosols and clouds, changes in stratospheric ozone, or for personal time in the sun at times when vitamin D can be produced. They were not compared with serum 25(OH)D concentrations for older adults living in various states. However, the results of this study should pave the way for additional studies incorporating such data from individuals.

## 5. Conclusions

This ecological study shows that the contribution of various risk factors for cancer in the US over 36 years has changed from where solar UVB doses were strongly and significantly inversely correlated with many cancers to where only about 10 cancers are inversely correlated, and to a lesser extent. Most notable among those cancers for which solar UVB is no longer identifiable as a risk reduction factor are colorectal and renal cancers, myeloma, and NHL. Dietary factors linked to diabetes and obesity, which previous ecological studies in the US did not consider, now look very important. Additional research is indicated to determine how the different cancer risk-modifying factors interact and how best to change public health policies that affect cancer risk directly or indirectly.

## Figures and Tables

**Table 1:** National average cancer incidence rates, 2016–2020 [21] for cancers with incidence and/or mortality rates inversely correlated with solar UVB doses [26] in ecological studies reported in 2006.

Cancer	Mean Rate (Cases/100,000/yr)	
	Males	Females
Bladder, urinary	37	9
Brain	9	6
Breast		133
Colorectal	42	32
Corpus uteri		28
Esophageal	9	2
Gastric	7	3
Hodgkin’s lymphoma	3	3
Laryngeal	5	1
Leukemia	19	11
Liver	11	4
Lung	64	34
Myeloma	8	5
Non-Hodgkin’s lymphoma	24	16
Oral cavity	20	7
Ovarian		10
Pancreatic	15	11
prostate	105	
Renal	24	12

**Table 2:** DNA-weighted UVB dose at Earth’s surface, by US state, July 1992. Adapted from a map from [13].

State	UVB Dose (kJ/m^2^)
Alabama	6.0
Alaska	
Arkansas	5.7
Arizona	9.0
California	7.5
Colorado	8.2
Connecticut	4.7
Delaware	4.7
District of Columbia	4.7
Florida	8.0
Georgia	7.2
Hawaii	
Idaho	6.0
Illinois	4.5
Iowa	4.7
Indiana	4.7
Kansas	6.3
Kentucky	5.8
Louisiana	7.5
Massachusetts	4.6
Maine	4.1
Maryland	4.7
Michigan	4.2
Minnesota	4.1
Missouri	6.5
Mississippi	7.0
Montana	4.7
North Carolina	6.6
North Dakota	6.2
Nebraska	5.1
New Hampshire	4.1
New Jersey	5.2
New Mexico	9.5
Nevada	8.5
New York	4.7
Ohio	4.7
Oklahoma	7.5
Oregon	5.2
Pennsylvania	4.5
Rhode Island	4.7
South Carolina	7.2
South Dakota	4.5
Tennessee	6.3
Texas	7.8
Utah	8.0
Virginia	6.0
Vermont	4.2
Washington	4.5
Wisconsin	4.5
West Virginia	5.2
Wyoming	6.0

UVB, ultraviolet-B radiation.

**Table 3:** Cross-correlation analysis, *r*, adjusted *r*^2^, *p* value.

Factor	DM	LCF	LCM	Obs	UVB
Alcohol	0.40, 0.14, 0.007	0.03 0.00, --	0.24, 0.03, 0.12	0.25, 0.04, 0.11	0.35, 0.10, 0.02
Diabetes		0.59, 0.33, *	0.84, 0.69, *	0.84, 0.69, *	0.13, 0.00, --
Lung cancer, F			0.88, 0.77, *	0.54, 0.28, *	0.14, 0.00, --
Lung cancer, M				0.75, 0.55, *	0.39, 0.13, 0.008
Obesity					0.11, 0.00, --

* <0.001; Alc, alcohol consumption, 2016; DM, diabetes rates for non-Hispanic white (NHW) males and females, 2016; LCF, lung cancer incidence rate, females; LCM, lung cancer incidence rate, males; NHW, 2016–2020 (M, males); Obs, obesity rates for NHW males and females, 2017–2019; UVB, solar ultraviolet-B on Earth’s surface in July 1992, adapted from [26].

**Table 4:** Regression results for cancer incidence rates for males, by US state, 2016–2020.

Cancer	Equation	*r*, Adjusted *r*^2^, *p* (*p*)
All	410 + (4.2 × Obs) − (7.3 × UVB)	0.58, 0.30, 0.002, 0.02
	350 + (4.8 × Obs)	0.49, 0.23, <0.001
	549 − (9.5 × UVB)	0.39, 0.13, 0.008
All less lung	420 − (7.34 × UVB) + (0.77 × LCM)	0.54, 0.26, 0.006, 0.01
	480 − (8.3 × UVB)	0.43, 0.16, 0.004
	370 + (0.89 × LCM)	0.39, 0.13, 0.008
Bladder	43 − (1.7 × UVB) + (1.5 × Alc)	0.72, 0.50, <0.001, 0.09
	48 − (1.9 × UVB)	0.70, 0.47, <0.001
	29 + (3.1 × Alc)	0.42, 0.16, 0.004
Brain	8.5 − (0.14 × UVB)	0.31, 0.08, 0.03
Colorectal	12 + (0.13 × LCM) + (0.70 × Obs)	0.82, 0.65, <0.001
	10 + (1.0 × Obs)	0.78, 0.61, <0.001
	24 + (0.28 × LCM)	0.73, 0.52, <0.001
Esophageal	5.7 + (0.048 × LCM) − (0.29 × UVB) + (0.69 × Alc)	0.77, 0.56, <0.001, 0.001, 0.006
	8.5 − (0.39 × UVB) + (0.039 × LCM)	0.71, 0.48, <0.001, <0.001
	11 − (0.44 × UVB)	0.57, 0.31, <0.001
	7.1 + (0.69 × Alc)	0.33, = 0.09, 0.03
Gastric	9.0 − (0.36 × UVB)	0.55, 0.29, <0.001
Larynx	1.3 + (0.058 × LCM)	0.57, 0.31, <0.001
Liver	4.5 + (0.66 × DM)	0.37, 0.12, 0.01
Lung	11 + (6.4 × DM)	0.84, 0.69, <0.001
	72 − (1.2 × UVB)	0.14, 0.000
Non-Hodgkin’s lymphoma	28 − (0.84 × UVB)	0.56, 0.29, <0.001
Pancreatic	18 − (0.40 × UVB)	0.55, 0.29, <0.001
Prostate	130 − (3.4 × UVB)	0.14, 0.15, 0.005
Renal	11 + (0.19 × LCM)	0.75, 0.55, <0.001
	3.6 + (0.66 × Obs)	0.74, 0.53, <0.001

Alc, alcohol consumption, 2016; DM, diabetes rates for non-Hispanic white (NHW) males and females, 2016; LCM, lung cancer incidence rate, NHW, 2016–2020, males; Obs, obesity rates for NHW males and females, 2017–2019; UVB, solar ultraviolet-B on Earth’s surface in July 1992, adapted from [26].

**Table 5:** Regression results for cancer incidence rates for females, by US state, 2016–2020.

Type	Equation	*r*, Adjusted *r*^2^, *p* (*p*)
All	440 − (9.8 × UVB) + (1.7 × Obs)	0.63, 0.37, <0.001, 0.06
	430 − (7.7 × UVB)	0.53, 0.26, <0.001
	360 + (2.5 × Obs)	0.34, 0.09, 0.03
All less lung	36 − (5.6 × UVB) + (0.94 × LCF)	0.63, 0.36, 0.006, 0.009
	430 − (7.7 × UVB)	0.53, 0.26, <0.001
	310 + (1.3 × LCF)	0.52, 0.25, <0.001
Bladder	9.5 − (0.50 × UVB) + (1.0 × Alc)	0.76, 0.56, <0.001, <0.001
	13 − (0.64 × UVB)	0.65, 0.41, <0.001
	5.5 + (1.5 × Alc)	0.59, 0.33, <0.001
	5.2 + (0.074 × LCF)	0.45, 0.18, 0.002
Breast	160 − (2.3 × UVB) − (2.2 × DM)	0.66, 0.41, <0.001, <0.001
	180 − (2.8 × UVB) − (0.91 × Obs)	0.65, 0.39, <0.001, <0.001
	150 − (2.4 × DM)	0.51, 0.24, <0.001
	150 − (2.5 × UVB)	0.47, 0.20, 0.001
	150 − (0.70 × Obs)	0.34, 0.10, 0.02
Colorectal	10 + (0.73 × Obs)	0.80, 0.63, <0.001
	21 + (0.21 × LCF)	0.53, 0.27, <0.001
Corpus uteri	38 − (1.9 × UVB)	0.72, 0.50, <0.001
Esophageal	2.2 − (0.13 × UVB) + (0.16 × Alc)	0.72. 0.49, <0.001, 0.02
	2.7 − (0.15 × UVB)	0.67, 0.44, <0.001
	1.1 + (0.29 × Alc)	0.47, 0.21, 0.001
	1.0 + (0.015 × LVF)	0.41, 0.15, 0.006
Gastric	4.1 − (0.14 × UVB)	0.38, 0.12, 0.01
Laryngeal	0.19 + (0.021 × LCF)	0.39, 0.13, 0.01
Liver	2.8 + (0.13 × DM)	0.36, 0.11, 0.02
Lung	43 + (3.2 × DM) − (2.7 × UVB)	0.75, 0.54, <0.001, <0.001
	30 + (1.1 × Obs) − (1.8 × UVB)	0.63, 0.36, <0.001, 0.02
	30 + (2.9 × DM)	0.59, 0.33, <0.001
	67 − (2.2 × UVB)	0.39, 0.13, 0.008
Non-Hodgkin’s lymphoma	19 − (0.60 × UVB)	0.51, 0.25, <0.001
Pancreatic	14 − (0.44 × UVB)	0.67, 0.44, <0.001
Renal	−2.0 + (0.46 × Obs)	0.84, 0.69, <0.001
	4.9 + (0.13 × LCF)	0.55, 0.29, <0.001

Alc, alcohol consumption, 2016; DM, diabetes rates for non-Hispanic white (NHW) males and females, 2016; LCF, lung cancer incidence rate, NHW, 2016–2020, females; Obs, obesity rates for NHW males and females, 2017–2019; UVB, solar UVB on Earth’s surface in July 1992, adapted from [26].

**Table 6:** Comparison of findings regarding solar UVB dose and cancer incidence between the present study and two ecological studies in 2006 [7,8].

Cancer	UVB (2016–2020)	UVB, Males [8] *	UVB, Females [8] *	UVB (2006), Males [7]	UVB (2006), Females [7]
Bladder	yes	1.13	1.15	yes	yes
Brain	M only	1.08	1.07		
Breast	yes		1.06	yes	yes
Cervical			0.84		no
Colon		1.11	1.14	yes	yes
Colorectal	no				
Corpus uteri	yes		1.49		yes
Esophageal	yes	1.27	1.07	yes	yes
Gastric	yes	1.42	1.27	yes	yes
Hodgkin’s lymphoma	no	1.16	1.19	yes	yes
Laryngeal	no	0.87	0.80	yes	yes
Leukemia	no	1.09	1.15	no	no
Liver	no	1.01	1.05	no	no
Lung	F only			no	no
Myeloma	no	1.19	1.22	no	no
Non-Hodgkin’s lymphoma	yes	1.08	1.09	yes	yes
Oral cavity	no	0.77	0.83	no	no
Ovarian	no		1.03		yes
Pancreatic	yes	1.09	1.17	yes	no
Prostate	yes	1.20		?	
Rectal		1.27	1.14	yes	yes
Renal	no	1.09	1.17	yes	yes

* A value greater than 1.00 indicates higher cancer rates at higher latitudes (lower solar UVB doses). F, females; M, males; UVB, ultraviolet-B radiation.

**Table 7:** Incidence rates for various cancer sites for 1982 and 2018 [2].

Cancer Site	M, Inc. Rate * 1982	M, Inc. Rate * 2018	M Inc. Rate *, Ratio 2018 to 1982	F, Inc. Rate *, 1982	F, Inc. Rate * 2018	F, Inc. Rate * Ratio 2018 to 1982
All	501	483	0.96	376	421	1.12
Breast				107	134	1.25
Colorectum	68	40	0.59	55	33	0.60
Corpus uteri				28	28	1.00
Liver	5	12	2.4	3	4	1.3
Lung & bronchus	94	51	0.54	36	43	1.2
Melanoma	12	34	2.8	11	20	1.8
Prostate	105	114	1.09			
Urinary bladder	37	31	0.84			

F, female; Inc., incidence; M, male; *, cases/100/000/year.

## Data Availability

The original contributions presented in the study are included in the article or in the references provided. Further inquiries can be directed to the corresponding author.
